# Micron-gap spacers with ultrahigh thermal resistance and mechanical robustness for direct energy conversion

**DOI:** 10.1038/s41378-019-0071-4

**Published:** 2019-07-15

**Authors:** Samuel M. Nicaise, Chen Lin, Mohsen Azadi, Tara Bozorg-Grayeli, Promise Adebayo-Ige, Drew E. Lilley, Yann Pfitzer, Wujoon Cha, Kyana Van Houten, Nicholas A. Melosh, Roger T. Howe, Jared W. Schwede, Igor Bargatin

**Affiliations:** 1 Mechanical Engineering and Applied Mechanics, University of Pennsylvania, Philadelphia, PA 19104 USA; 2 Materials Science & Engineering, Stanford University, Stanford, 94305 CA USA; 3 Chemical and Biomolecular Engineering, University of Pennsylvania, Philadelphia, PA USA; 4 Spark Thermionics, Berkeley, CA 94720 USA; 5 Electrical Engineering, Stanford University, Stanford, CA 94305 USA

**Keywords:** Electrical and electronic engineering, Electronic properties and materials, NEMS

## Abstract

In thermionic energy converters, the absolute efficiency can be increased up to 40% if space-charge losses are eliminated by using a sub-10-µm gap between the electrodes. One practical way to achieve such small gaps over large device areas is to use a stiff and thermally insulating spacer between the two electrodes. We report on the design, fabrication and characterization of thin-film alumina-based spacers that provided robust 3–8 μm gaps between planar substrates and had effective thermal conductivities less than those of aerogels. The spacers were fabricated on silicon molds and, after release, could be manually transferred onto any substrate. In large-scale compression testing, they sustained compressive stresses of 0.4–4 MPa without fracture. Experimentally, the thermal conductance was 10–30 mWcm^−2^K^−1^ and, surprisingly, independent of film thickness (100–800 nm) and spacer height. To explain this independence, we developed a model that includes the pressure-dependent conductance of locally distributed asperities and sparse contact points throughout the spacer structure, indicating that only 0.1–0.5% of the spacer-electrode interface was conducting heat. Our spacers show remarkable functionality over multiple length scales, providing insulating micrometer gaps over centimeter areas using nanoscale films. These innovations can be applied to other technologies requiring high thermal resistance in small spaces, such as thermophotovoltaic converters, insulation for spacecraft and cryogenic devices.

## Thermionic converters: insulated micron-scale gaps

An aluminum oxide spacer effectively insulates one electrode from another in a thermionic device. Energy conversion devices such as thermionic and thermophotovoltaic devices require thermal and electrical insulation across two electrodes separated by a small space. However, finding materials that can effectively occupy this gap and minimize parasitic heat flow is challenging. Now, a team led by Igor Bargatin from University of Pennsylvania demonstrate that deposited aluminum oxide spacers can be integrated between electrodes in a thermionic device, achieving a thermal conductivity in a vacuum of just 5 mW m^–1^ K^–1^—below that of aerogel—and can sustain compressive stresses of 0.4–4 MPa while avoiding electrical or thermal shorts. It is found that only 0.2–0.5% of the surface area of the spacer is conducting heat, and thermal conductivity is nearly independent of both spacer thickness and gap distance.

## Introduction

**Fig. 1:**
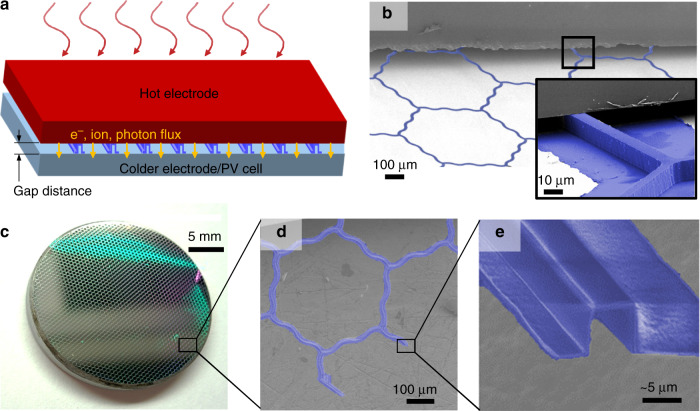
Overview of thermal spacer design. **a** Diagram of two hot electrodes in a thermionic or thermophotovoltaic converter separated by the spacer (not to scale). **b** Scanning-electron micrograph (SEM) showing how the spacers maintain a gap between planar substrates at a distance defined by the spacer height. **c** The released spacer samples are modular and can be placed on any chosen substrate, such as a polished molybdenum electrode. **d** SEM of the U-beam ribs and hexagonal honeycomb pattern (in this case, the wavy design with low curvature) of the spacer. **e** Zoomed-in SEM showing the cross section of a spacer rib. The film thickness is about 400 nm and spacer height about 6 μm. The spacers in the SEMs are false-colored (blue) to enhance contrast

In high-temperature solid-state energy converters, optimizing the spacer that separates the hot components from the cold (Fig. 1a) can greatly boost the conversion efficiency. For example, in thermionic converters with micron-gaps or photon-enhanced emission, conversion efficiencies beyond 40% can be achieved in vacuum as long as the spacers offer negligible conduction loss^1–4^. Similarly, in thermophotovoltaics the performance can be significantly increased through near-field thermal radiation across vacuum gaps of a several hundred nanometers or less^5–8^. However, using such small gaps is challenging because the spacer must be simultaneously thin enough to minimize thermal conductance and strong enough to prevent thermal or electrical shorting due to the spacer bending or collapsing^1,9^.

Heterogeneous materials could in principle be used for spacers if they offer much lower thermal conductance than the least conducting uniform materials, such as amorphous solids, which typically have *k*~1 Wm^−1^K^−1^ ^10,11^. Nanolaminates can reach a conductivity of *k*~0.1 Wm^−1^K^−1^, significantly below the conductivity of the constituent alternating nano-meter thin layers^12–16^. Aerogels, with their ultralow density and nanoscale porosity, provide even lower conductivities of 0.01–0.03 Wm^−1^K^−1^ in vacuum^17–23^. Considering structures as well as just materials, multilayer insulator assemblies made from thin reflecting films can achieve effective conductivities *k*_*eff*_ below 10^−4^ Wm^−1^K^−1^ at cryogenic temperatures^24–27^. However, both aerogels and multilayer insulator assemblies are not generally strong enough to sustain any practical compressive stress between thermophotovoltaic or thermionic converter electrodes. In addition, both aerogels and multilayer insulators are normally continuous sheets and not easily patterned with holes to allow for the transmission of electrons or photons.

Therefore, to sustain inter-electrode insulation and micron-scale spacing, researchers previously used sparse and small-area microstructures in thermionic and thermophotovoltaic devices. Some small-scale area devices (< 1 mm^2^) were only designed for radiative heating and therefore could not sustain significant compressive force^3,28,29^. For some centimeter-scale devices, spacers were designed around the perimeter of the electrodes^30–32^, but such device architectures were prone to shorting due to bowing or surface roughness at the unsupported center of the electrode. To prevent shorting and achieve larger-area micron-gap converters, spacers should be distributed over the entire electrode area. Such distributed spacers were recently implemented in thermionic converters using both randomly distributed beads^33^ and arrays of microfabricated columns^34^, but both architectures still reported low conversion efficiencies. In addition, DiMatteo et al. microfabricated tubular spacers directly into top of one of the electrodes, which provided adequate thermal insulation in thermophotovoltaic experiments^35^, though our calculations suggest that they are too flexible to prevent shorting at >100 kPa compression. We also note that these spacers were microfabricated directly into one of the electrodes^34,35^; therefore, such designs are limited to using only electrodes that can serve as microfabrication substrates, potentially reducing the performance. In addition, because of close thermal contact with the substrate electrode, they do not take advantage of additional contact resistance such as in spacers that are fabricated separately and then stacked with the electrodes to build a device.

To address these challenges, we present a microfabricated spacer design with nanoscale thickness that simultaneously provides robust micron-scale gaps, high thermal insulation, easy scaling to macroscopic areas, and the ability to function on any substrate. As depicted in Fig. 1, our spacer is a hexagonal mesh of ribs that are made of insulating atomic-layer-deposited aluminum oxide (ALD alumina). The mesh is sparse enough to allow for electrons, photons, or other particles to freely pass from one electrode to the other but is interconnected in-plane so that it can be manually transferred from a sacrificial mold to almost any electrode material/substrate (i.e., offers versatile modularity). Because the spacer was not directly fabricated into an electrode, the additional spacer/electrode interface increases the overall thermal resistance. As is detailed in the results section, we measured an effective thermal conductivity of ~5 mWm^−1^K^−1^ in vacuum (see Supplementary Section S6), lower than that of aerogels (although, if the materials were compared for non-vacuum performance, the conductivity of aerogels would be lower than that of the spacers). Surprisingly, the conductance was independent of variations in the spacer height or plate thickness and instead defined by the sparse contact points that limited the conducting cross-section to <1% of the ribs. We found the spacers to be compliant and robust with respect to in-plane tension, showing no apparent damage when we applied in-plane strains of 5–10%, and strong under out-of-plane compression, withstanding 0.4–4 MPa of pressure before failure.

## Design

As pictured in Fig. 1a, the emitter of a thermionic or thermophotovoltaic converter is typically at a temperature of >1000 °C and in close proximity to the cooler collector at a temperature of ~600 °C or lower. As a result, significant heat loss can occur between these two electrodes, reducing the efficiency. The conductive heat flow from the emitter to collector can be written as $\dot Q_{\mathrm{c}} = \frac{{k_{{\mathrm{eff}}}S}}{d}\left( {T_{\mathrm{E}} - T_{\mathrm{C}}} \right)$, where *S* is the total surface area of the overlap between the emitter and collector (rather than just the cross section of the sparse spacer), *d* is the inter-electrode gap distance, *T*_E_ and *T*_C_ are the temperatures of the emitter and collector, respectively, and *k*_eff_ is the effective (average) conductivity of the physical spacer that separates the electrodes. To reduce the parasitic heat loss through the spacer, *k*_eff_ must be minimized, since the other parameters, *T*_E_, *T*_C_, *d*, and *S*, are typically dictated by efficiency optimization and operational requirements. Given the typical power output density of 10–100 Wcm^−2^ for thermionic converters, a temperature difference of ~400 K, and *d* of a few microns, the *k*_eff_ should be ~1 mWm^−1^K^−1^ to avoid > 10 Wcm^−2^ being parasitically lost through the spacer.

Such low conductance values are quite challenging to achieve. For example, Belbachir et al. fabricated sub-mm^2^ SiO_2_ columns into substrates, with the columns spaced with a period of several millimeters to minimize heat conduction^34,36^. Yet, the columns still allowed a thermal conductance of ~300 mWK^−1^cm^−2^ from the emitter to collector, which leads to heat losses larger than the typical power output of a thermionic converter and is, therefore, unacceptable for efficient conversion. Ito et al. provided an example a spacer architecture comprised of sparse and small (a few micrometers on each side) silica columns^37^. While the array provided an effective thermal conductivity that would be low enough, their setup did not test for thermal and electrical shorting. Furthermore, based on our experience, the sparse, rigid columns dig into thermionic electrodes during thermal cycling by hundreds of degrees, causing damage and shorting. Thus, high-temperature spacers need to be distributed more evenly, and their conductance needs to be reduced by designing them to be thinner and less thermally coupled to the electrodes.

Our spacer design aims to minimize the heat conduction while also providing a mechanically robust and modular architecture that can be adapted for future research and improvements. As shown in Fig. 1c, d, the design has hexagonal symmetry, with thin ribs connected to form transparent openings. For mechanical robustness, we used a “U-beam” design (Fig. 1e) for the ribs that provides enough mechanical strength to ensure the desired gap between the electrodes. The ribs were made with relatively thin (100–800 nm) walls to limit thermal conduction. Amorphous ALD alumina provided intrinsically high electrical and thermal resistance (thermal conductivity of about 2 Wm^−1^K^−1^)^38^.

The critical in-plane and out-of-plane mechanical characteristics were optimized through finite element simulations. (Details on COMSOL modeling are given in Supplemental Section S1.) The simplest array of connected ribbing was a straight-leg hexagonal unit cell, as shown in Table S1. However, we found that this design often failed during experimental heating due to its limited ability to accommodate in-plane deformations. During converter thermal cycling from room temperature, the hotter emitter (at up to 2000 K) expands and forces up to ~1% in-plane strain on the spacer array. This is larger than the typical failure strain of brittle solid materials and on the same order as the largest strains that ALD alumina can sustain without fracture. In order to accommodate the thermal strain during the thermal cycling, we designed and simulated four other unit cells with increased in-plane compliance, as shown in Table S1: wavy ribs with high or low curvature and expanding crableg flexures with hexagonal or square symmetry. All these designs use geometry to accommodate in-plane deformations while still remaining stiff with respect to out-of-plane compression. In experiments, these wavy and expanding spacers were more stretchable and mechanically robust than the straight hexagonal spacers, allowing them to better accommodate large thermal strains.

Despite the thin-walled nature of the ribs, we found that the array of U-beam ribs was sufficiently stiff in compression to maintain the desired inter-electrode gap. When simulating a compressive pressure of 100 kPa, a hexagonal spacer with a rib height of 8 μm and film thickness of 800 nm deflected just ~1 nm, corresponding to an effective simulated modulus of ~3 GPa. We note, however, that the experimentally measured modulus was ~100 times lower due to imperfect mechanical contact between the spacer and electrodes, as discussed below.

**Fig. 2:**
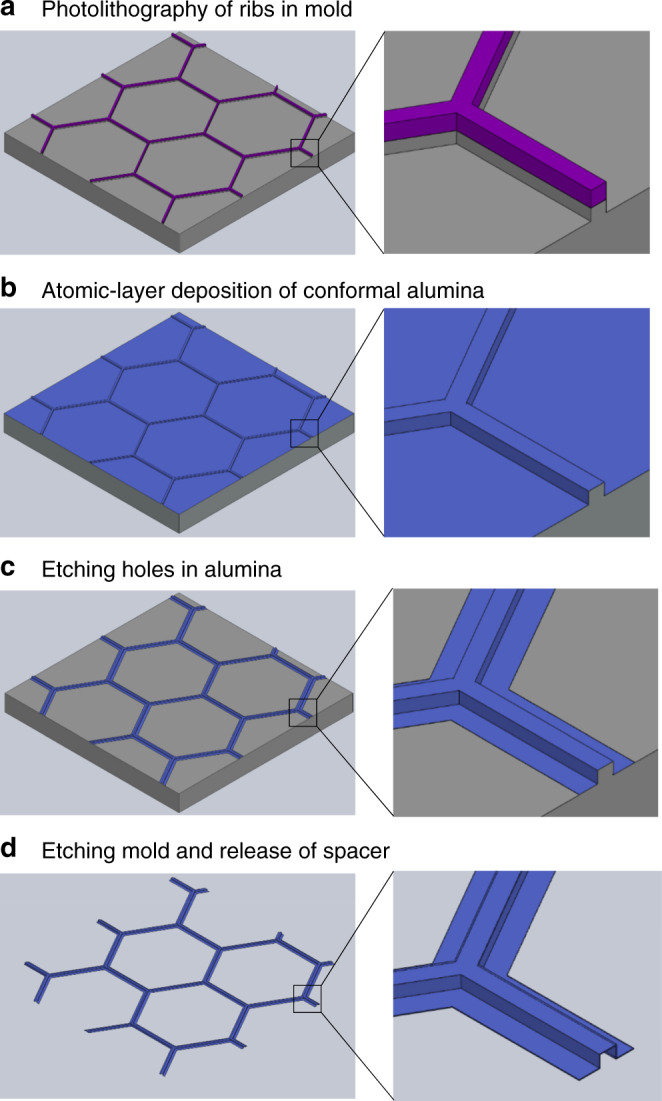
Schematic of the fabrication process. **a** Photolithography is used to etch ribs of a few micron width in the surface of a silicon sacrificial substrate, and, **b** after coating and **c** etching openings in a thin atomic-layer deposited film of alumina, **d** the spacer is lifted away from the substrate by undercutting the silicon with an isotropic XeF_2_ etch. In the figure, magenta is the photoresist, blue is the ALD alumina, and grey is the silicon/mold

The microfabrication process (based on the previously reported process for honeycomb plates^39^) is presented in Fig. 2 and is detailed in the Materials and Methods section and Supplemental Section S2. Briefly, inter-connected ribs were etched into silicon wafers with photolithography and conformally coated with alumina via atomic layer deposition (ALD). We etched the openings in the alumina with a second photolithography step and finally released spacer samples by XeF_2_ vapor etching the sacrificial silicon mold. The planar lithography method allows the process to scale to spacer areas of many square centimeters with no adaptations. Although we focused on ALD aluminum oxide spacers in this study, any other conformally deposited insulating materials or composites can be used instead to further improve the spacers or adapt them to other applications. Finally, since the spacers were patterned on a sacrificial silicon mold instead of directly on an electrode, they could be manually transferred to a substrate with any other shape or material. Figure 1c is an example of this transfer, where a spacer was placed on top of a molybdenum electrode. Additional discussion of the ease of fabrication and sample handling is included in Supplemental Section S2.

## Mechanical compression results and discussion

By stacking the spacer between two chips of silicon wafer (Fig. 1b), we typically observed a gap that was within 1 μm of the nominal spacer height (using the capacitance measurement setup described in more detail in Supplemental Section S4). However, further testing of the spacer design was necessary to demonstrate that they would be strong enough to support larger mechanical loads that are typical of practical applications. Researchers typically test the mechanics of nano and micron-scale architectures with specialized tools, such as a nanoindenter, that apply small forces over tiny areas. In contrast, our testing required much higher forces and larger areas because of the unusually high compressive strength and size of the spacers. For this reason, we used a standard Instron material tester (Figure S2) that provided loads up to 2kN, though we had to carefully ensure the pressure was applied evenly over the entire spacer sample (Supplemental Section S3).

**Fig. 3:**
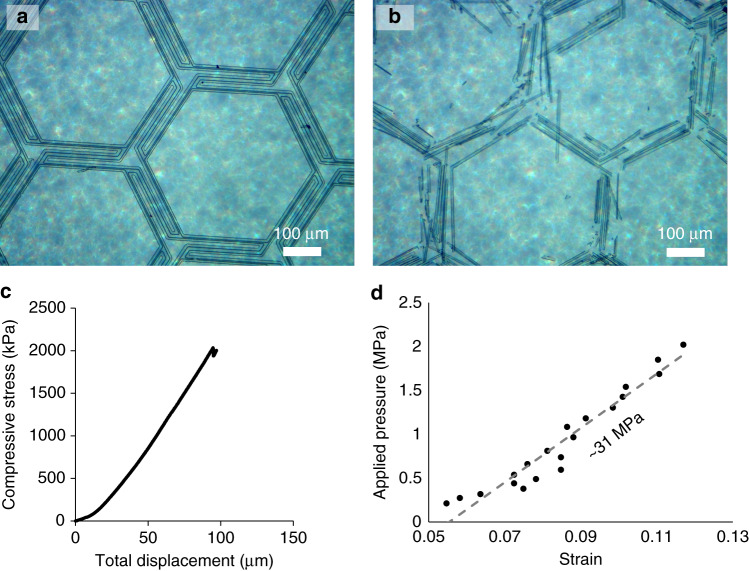
Images and graphs from the compression testing. **a** Microscope image of an expanding hexagonal spacer before compression and **b** microscope image of the same sample after complete failure. **c** Stress vs. displacement curve from the Instron material tester for an expanding hexagonal sample, similar to (**a**, **b**). Note that large displacements measured by the materials tester are due to the deformations of the load cell and the test stand (see Supplementary Section S3). The spacer itself generally deformed by <1 μm, as determined from the capacitive measurements of the inter-electrode gap. **d** Stress vs. strain curve for a different sample of the wavy low-curvature design. All examples are for *d* = 4.2 μm and *t* = 800 nm

The measured failure strengths of various samples were all at least several atmospheres (see Supplemental Section S3 for full details and results), ranging from 0.39 MPa for 200-nm-thick spacers to almost 4 MPa for the 800-nm-thick spacer. Figure 3a, b provide example microscope images of pristine (before) and crushed (after) spacers. Figure 3c provides an example of the applied stress and failure of one sample. This high measured failure strength was surprising for such sparse structures, considering that they had a nominal volume density of 0.37–3.2% and mass density of 0.014–0.126 gcm^−3^, which is similar to aerogels. The strength of our spacers can be attributed to the alumina material and U-beam rib design, both of which are known to be stiff and strong with respect to transverse loads. In contrast, inorganic aerogels of similar density and porosity have a random geometry and are therefore weaker, with yield strengths of <1 MPa and compressive moduli of <10MPa^40–42^. Similarly, thin blankets of multilayer insulator assemblies cannot be compressed with any force greater than gentle manual handling without risking a thermal short.

Using the capacitance measurement setup (Supplemental Section S4), we determined the compression displacement with a precision of < 100 nm and confirmed that inter-electrode gaps were typically within 1 μm of nominal spacer heights. For the shortest spacers, we could readily achieve gaps of 3 μm or less, providing a route to high-efficiency thermionic energy conversion with microgap architectures. Figure 3c, d shows typical stress-displacement curves that we obtained for the stiffest samples. The low-end estimation of the effective compressive modulus, at ~31 MPa, is ~100 times less than predicted by COMSOL simulation, suggesting that only about 1% of the spacer was in contact with the electrodes for small deformation, which also has important implications for thermal conduction, as discussed below.

## Thermal Characterization Results and Discussion

**Fig. 4:**
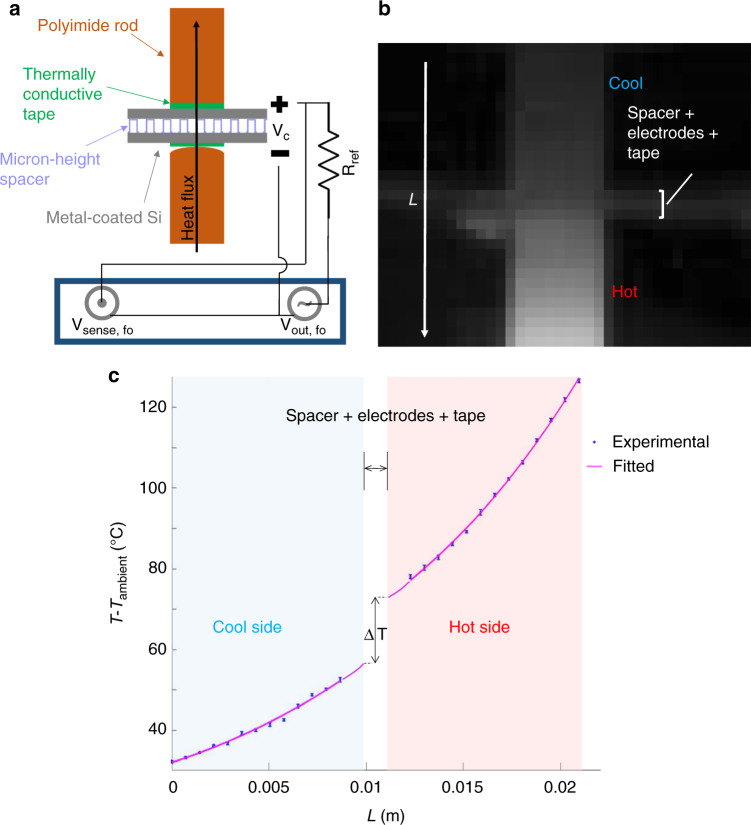
**a** Schematic of the modified meter-bar thermal measurement setup (not to scale). **b** Example thermal infrared image of the meter bars, electrodes and spacer. Heat flows from the bottom (hot side) to the top (colder side). **c** Extracted temperature profile from (**b**). The blue asterisks are the experimental temperature measurements and the magenta solid curve is the non-linear fitting. The temperature data points near the interface were not used in the fitting due to low thermal emissivity of the electrodes. To reduce noise, we averaged ~1000 video frames for each thermal measurement. Vertical error bars are one standard deviation and horizontal error bars are ~0.6 mm (one pixel)

We measured the transverse thermal conduction of our spacers using an adapted meter-bar technique with optical readout of temperature (Fig. 4a, b and S4). We ignored convection and conduction by air gap because we were testing in high vacuum (typically evacuated to < 3 × 10^−6^ Torr). As detailed in the Methods and Materials section and Supplemental Section S5, our setup used custom polyimide meter bars, resulting in non-linear temperature distribution along the rod length (instead of the linear distribution typical for copper meter bars) due to the non-negligible radiation from the sides of the polyimide rods. The thermal conductance between the electrodes was determined from the measured temperature drop: $C_{{\mathrm{thermal}}} = \left| {q\prime\prime /\Delta T_{{\mathrm{drop}}}} \right|$, where $q\prime\prime = - \kappa _{{\mathrm{polyimide}}}\frac{{dT\left( y \right)}}{{dy}}|_{{\mathrm{interface}}}$ and *κ*_polyimide_ is the polyimide thermal conductivity. As is evident in Fig. 4b, it was not possible to directly determine $\frac{{dT\left( y \right)}}{{dy}}|_{{\mathrm{interface}}}$ from the thermal camera image because the silicon electrodes had a different emissivity compared to polyimide and each single pixel of the camera covered much more than the interface. In order to explicitly determine the temperature gradient at the material-material interfaces, $\frac{{dT\left( y \right)}}{{dy}}|_{{\mathrm{interface}}}$, we simultaneously fit the experimental temperature profiles from the hot and cold polyimide rods, as shown in Fig. 4c. These fitted lines were extrapolated towards each other to specifically calculate the temperature drop across the non-polyimide stack: the thermal tape, silicon electrodes, and spacer gap.

From the fitted temperature profiles, we obtained thermal conductance measurements for over 50 spacer samples with *d* = 3–8 μm and *t* = 200–800 nm at varying compressive pressures. Generally, the thermal conductance varied between 10 and 30 mWcm^−2^K^−1^. For comparison, the expected radiation conductance through the gap is < 10^−1^ mWcm^−2^K^−1^, confirming that thermal conduction is the dominant heat transfer mechanism in our measurements. The measured conductance values compare favorably (i.e., within an order of magnitude) to the results by DiMatteo et al.^35^, which is surprising considering our mesh spacer covers the entire electrode and is not just individual pillars. At the same time, our spacers were substantially stiffer than the DiMatteo spacers, which decreases the risk of thermal and electrical shorts.

**Fig. 5:**
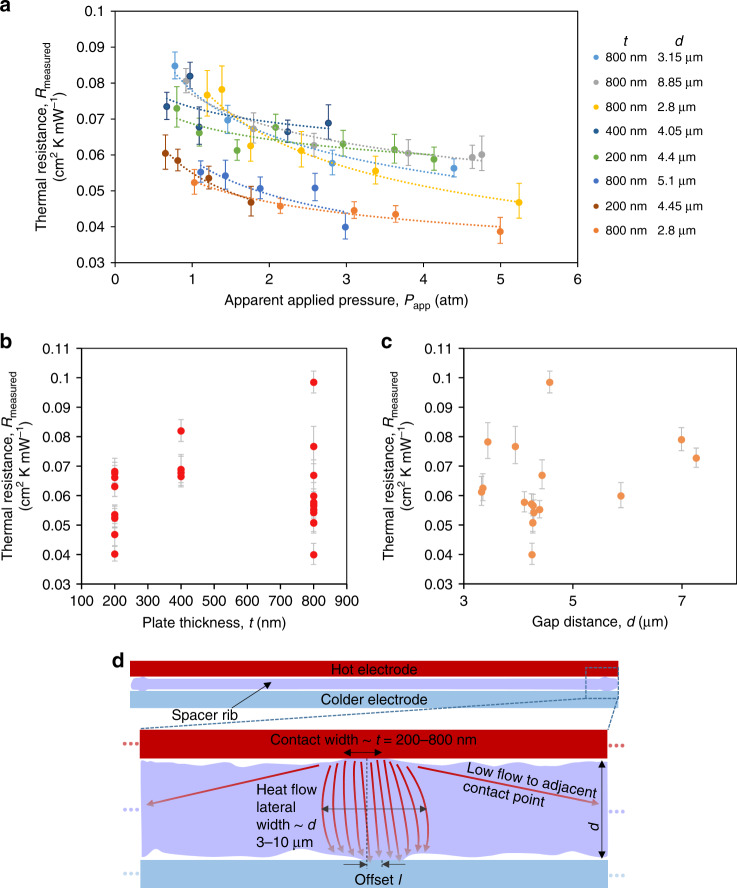
Thermal resistance measurement results. **a** Graph of thermal resistance at increasing applied pressure for many different spacer samples. For each series of data, the dashed lines represent the ${P}_{eff}^{ - 0.94}$ scaling predicted by the theory of contact thermal conductance^43^. The horizontal range was 1% based on the verified specification of the force sensor. **b** Graph of thermal resistance vs. thickness of the ALD alumina with a spacer gap *d* of 4–6 μm. **c** Graph of thermal resistance vs. gap distance for a spacer thickness *t* of ~800 nm. The data for (**b**, **c**) are for spacers with the expanding hexagon design at an applied pressure of 100–300kPa. The horizontal error bar for (**b**) is 5% based on experimental variation in the deposition process. **d** Schematic of model for the heat flow through a single paired point contact in the spacer rib. (top) The spacer only contacts the top and bottom electrodes at small points that are sparse along the length of the rib. (bottom) The heat flow is primarily between proximal opposite contacts points in the top and bottom of the spacer. The area of the contact points is modeled to have side lengths similar to the thickness (*t*) of the alumina film, and the width through which the heat flows is estimated to be similar to the spacer height ***d***

We characterized the thermal resistance for the expanding hexagonal design for varying applied pressure *P*_app_, gap distance *d*, and plate thickness *t* (Fig. 5a–c). The experimental measurements showed a clear dependence on *P*_app_, as expected for structures where contact thermal resistances play a major role^43^. Specifically, increasing compressive pressure *P*_app_ monotonically decreased *R*_measured_ until electrical short at 100–500 kPa. We note that the pressure *P*_app_ is reported based on the total area of the overlapped electrodes, not just the spacer ribs (i.e. we include both the areas of the openings and ribs). However, while we anticipated the thermal resistance of the spacers, *R*_measured_ = 1/C_thermal_, to increase with the increasing gap *d* and decreasing thickness *t*, the experimental results did not reveal any obvious trends with *d* and *t*. As shown in Fig. 5b, c, the thinnest samples often showed resistances similar to thicker and taller samples. Nonetheless, all tested spacers, regardless the film thickness and range of heights, provided a high degree of thermal insulation—a finding that provides significant latitude for how energy converters are designed and operated.

Moreover, the spacers conducted much less heat than expected from a back-of-the-envelope calculation. Naively, one could assume that the spacer ribs are in perfect contact with the electrodes, meaning that *R*_measured_ is mainly attributed to thermal conduction through the spacer vertical walls. Therefore, the “naive” thermal conductance can be estimated as $C_{{\mathrm{naive}}} \approx k_{{\mathrm{alumina}}}\frac{{FF}}{d}$, where the thermal conductivity of ALD alumina *k*_alumina_ = ~2 W/(m K)^38^ and the areal fill factor of the spacer ribs *FF* = ~0.217–1.894%. Assuming *d* ≈ 3–8 μm we obtain *C*_naive_ on the order of 10^2^–10^3^ mWcm^−2^K^−1^, one to two orders of magnitude larger than in our experimental measurements (Fig. 5a).

### Sparse contact point model

As detailed in the Supplemental Section S6, we developed a model to describe the thermal conductance that combines contact resistance from non-uniform asperities with a decrease in conducting pathways attributed to sparse contact points. Previous theoretical modeling shows that locally distributed asperities prevent solid surfaces from coming into perfect contact, even with high pressure pushing them together. As an example, the ratio of the actual contact area to the nominal contact for the majority of metallic joints does not exceed 1–2% even if the surfaces are polished and the contact pressure is on the order of 10 MPa^44^. In order to characterize the additional temperature drop related to a single constriction—the point at which two asperities from opposite solid surfaces allow conduction—the concept of thermal constriction resistance *R*_c_ was introduced: *R*_c_ = (*T*–*T*_0_)/*q*, where *T*_0_ and *T* are the required temperature differences for heat flow *q* with and without the existing constriction. Previous reports provide solutions to this problem by modeling the constriction as a simple circular disk in half space, accounting for the radius of the disc and the thermal conductivities of the two materials^43,45–47^. The total contact resistance, *R*_contact_ can be calculated as the parallel array of constrictions *R*_c_ in a given local area and, in the case of our spacers, accounts for roughly 1/3 of the total measured thermal resistance from the fits of Fig. 5a (see Figure S5).

The remaining thermal resistance was associated with the conductance through the spacer, *R*_spacer_ ~3.76 × 10^−2^ to 6.51 × 10^−2^ cm^2^K mW^−1^. As discussed in Supplemental Section S6, this data can be used to calculate the effective thermal conductivity *k*_eff_ of the spacer material itself, which is ~ 5 mWm^−1^K^−1^, or less than most aerogels. We note, however, that aerogels hypothetically also have significant contact resistance and therefore may be overall more or less conducting than the spacers. *R*_spacer_ was about an order of magnitude higher than the “naive” resistance estimated above, 1 × 10^−3^ – 1 × 10^−2^cm^2^KmW^−1^. As depicted in Fig. 5d, we believe that the discrepancy is due to the imperfect thermal contact between the spacer film and the electrodes: thermal contact points are sparsely distributed along the rib walls. If the contact points on the top and bottom are not perfectly aligned in-plane, a pressure of ~100 kPa is sufficient to force a more nearby asperity into contact (see Supplemental Section S6). Given that we generally use even larger applied pressures, we assumed that heat is conducted predominantly through paired contact points that were immediately opposite each other on the top and bottom, and the small conduction to adjacent contact points was calculated to be negligible.

We calculated the nominal heat resistance through the rib at a single paired contact to be $R = \frac{d}{{\kappa A}} = \frac{d}{{\kappa td}} = \frac{1}{{\kappa t}}$, where *κ* is the thermal conductivity of the spacer material. We found that *R* = 625–2500 KmW^−1^ per contact point for *t* = 200 or 800 nm, and given the experimentally measured *R*_spacer_ ~3.76 × 10^−2^–6.51 × 10^−2^cm^2^KmW^−1^, we estimate that the density of paired contact points is 11,500–63,300 per cm^2^, equivalent to 20–110 points per hexagon unit. In other words, considering that the expanding hexagon design had ~2370 mm of ribbing sidewall per square centimeter, there was a paired contact point every 20–120 μm, or 1–7 points per hexagon side.

This contact density is consistent with our experimental observations of surface roughness created by the plasma etching process (Figure S4). The model predicts thermal resistances many orders of magnitude larger than that for the naive model in which the spacer is in perfect thermal contact with the electrodes on both sides. Furthermore, the model accounts for the experimental lack of dependence on *d* and *t*. The influence of the spacer height is eliminated because conduction through each contact point happens through a width also approximately equal to *d*. While the contact point resistance is dependent on *t*, (see calculation in previous paragraph), the stiffness of the ribs is also dependent on *t* (see Supplemental Figure S6). Therefore, more conducting points make contact for a given pressure when the film is thinner, explaining how the dependence on *t* is negated.

Our asperity contact point model further predicts that the measured compressive modulus should scale roughly linearly with the rib contact point area, and therefore be much smaller than the modulus naively predicted by assuming perfect contact on both sides. As detailed previously, the measured effective compressive elastic modulus was about 1% of the simulated value, whereas the effective thermal contact area was 0.1–0.5%. Therefore, both the mechanical stiffness and the thermal conduction data are consistent with similarly sparse distribution of contact asperities. While further testing is required to fully understand the connection between thermal conduction and compressive modulus, our sparse contact model provides a significant insight into the heat conduction through microgap spacer insulators that are fabricated separately from electrodes. Specifically, the model describes how the heat conduction can be independent of spacer height and thickness, and much lower than that in spacers monolithically fabricated into the electrodes. This highlights the remarkable characteristic of our spacers to provide multiscale functionality – that is, device areas on the centimeter scale, unique mechanical properties and inter-electrode gaps based on ribbing designs at the micrometer scale, and thermal insulation based on thin films and sparse contact points at the nanoscale. This investigation sets the foundation for future engineering of nonuniform sparse contact points in thin film microarchitectures to provide a tailored thermal resistance and mechanical stiffness for a variety of applications.

## Conclusion

We developed a modular, thermally insulating spacer for thermionic converters and other devices that require sub-10-μm separation between two planar surfaces at elevated temperatures. The spacers were comprised of ALD alumina, imparting low thermal conductivity and high out-of-plane compression robustness, including failure strengths of greater than 1 MPa. The spacer designs incorporated in-plane compliance to allow for thermal expansion strains of ~1% without failure. The thermal conductance of the spacers was measured in an experimental setup that simulated a thermionic or thermophotovoltaic converter. Thermal conductance was independent of spacer gap distance and alumina plate thickness, measuring 10–30 mWcm^−2^K^−1^, which is over an order of magnitude better than most previous reports of robust spacers. A sparse contact point conductance model was used to determine the thermal resistance of the spacer structure and the contact resistance of the imperfect contacts between the alumina surfaces and sandwiching electrodes. About one third of the total thermal resistance was attributed to the interface contact. The effective thermal conductivity from the spacer structure itself was ~5 mWm^−1^K^−1^, smaller than the most insulating aerogels. In this paper, we focused on the case of thermionic energy conversion, though we anticipate the presented spacer design to be useful in thermophotovoltaics, cryogenics, pyroelectric devices, spacecraft, and other applications requiring micrometer isolation between different temperatures and voltages, especially if there is a need for openings that allow the free flow of particles, i.e., ions, gas molecules, photons, or electrons.

## Materials and methods

Based on the design considerations and finite-element simulations, we fabricated spacer samples with all four wavy and expanding designs, plate thicknesses *t* of 200–800 nm and spacer height *d* of 3–8 μm. We performed mechanical compression testing to characterize the failure strength and compressive modulus. We determined the thermal conductance of samples by analyzing the externally read temperature distribution through the meter bar test setup in a home-built vacuum chamber.

### Spacer microfabrication

The first steps were based on the previous fabrication process^39^. Front-side patterning of a silicon wafer (photolithography and deep reactive ion etching) created the rib-patterned mold that was then conformally coated with aluminum oxide using atomic layer deposition at 250 °C with tetra methyl aluminum and water vapor (Cambridge NanoTech S200, 5 s delay per pulse). The rib height was measured with a KLA-Tencor P7 2D profilometer. The thickness was measured with a Filmetrics F40 broad spectrum spectrometer. The next step was to remove the alumina between the ribs to create the openings for electrons. In order to create etch masks that protected the ribs, we spin-coated MICROPOSIT SPR220-7.0 resist (Dow Chemical Company) to a thickness of 6–10 μm, performed i-line exposure on a Suss MA-6 mask aligner, and developed for 5–10 min in room temperature MICROPOSIT MF 26A developer. The alumina was etched in either an Oxford Instruments Cobra or a Plasma-Therm Versaline inductively coupled plasma RIE with BCl_3_ (and Cl_2_ for the Versaline) at 100 W. The resist was removed with sonication in acetone, by rinsing with acetone, methanol, and isopropanol, and then oxygen plasma cleaning (100 W for 10 min). Finally, the spacer arrays were released by isotropically etching the silicon mold with XeF_2_ vapor in a Xactix/SPTS e1 system for 200–500 cycles that lasted 70 s with a gas pressure of 2 T. After release, the samples could be handled with a tweezer and manually transferred to another substrate. Furthermore, they were robust enough that they could provide the as-designed thermal insulation and gap separation after packaging, commercial shipping, and storage for over 1 month.

### Microscopy & Imaging

Optical imaging was taken with standard bright-field optical microscopy on a Zeiss Axio Imager M2m. Scanning electron microscopy was carried out on a JEOL 7500F high resolution SEM with the off-angle secondary-electron detector at an acceleration energy of 2–10 kV and working distance of 4–6 mm. Atomic force microscopy (AFM) was performed after the samples were cleaned using acetone and isopropyl alcohol with ultrasonic cleaning and rinsed with DI water followed by N_2_ blow dry. The AFM measurements were done using a table top Icon Bruker Dimension 3000 AFM with tapping mode in air at room temperature and room relative humidity. The scan frequency was set to 1 Hz. Drive amplitude and gain were manually set to achieve the highest resolution for ultraflat samples. The images were processed using Gwyddion freeware, version 2.5^48^. Midplane subtraction was used to level the data and the rows were aligned using median method.

### Mechanical Compression Testing

In order to experimentally validate the stiffness of the spacers to out-of-plane compression, we characterized the failure strength of many samples via compressive loading in a standard Instron 5564 materials tester (Figure S2). The spacer samples were stacked between a thick slab of glass, on bottom, and a tungsten-coated chip of a silicon wafer and another 0.5 mm thick glass chip, on top. The compression was video recorded from underneath during the testing with a generic handheld computer-connected microscope to determine the morphology and failure of the spacer at varying loads. Since the materials tester is traditionally intended for macroscale materials, we could use load cells rated for 2kN of force. The compression was applied over the course of several minutes, cyclically at first with a force that did not obviously damage the sample, and then to a higher force until catastrophic failure. The force measurement was precise enough (~0.5% error) to show a monotonic increase during the loading until a characteristic abrupt drop in the force reading at the same time as the catastrophic failure was observed on the microscope.

In order to achieve a higher accuracy for the measured displacement (the materials tester was precise to only ~20 μm), we measured the capacitance of the electrode-spacer-electrode parallel plate capacitor (Figure S3 and Supplemental Section S4). The bottom plate was a thick glass slab sputter-coated with > 100 nm of indium-doped tin oxide to provide an electrically conducting film. The top electrode, a mirror-polished disk of molybdenum, was suspended hanging from the compressor tip and lowered slowly onto the spacer sample to avoid premature damage.

### Thermal Conductance Testing

In order to measure the thermal conductance across the inter-electrode gap, we employed a meter bar technique based on the ASTM test^49^. Researchers have modified the relevant ASTM Standard D5470 in numerous ways to fit special conditions^50–54^. We also needed to do so to characterize our highly insulating spacers. While copper rods are typically used for the meter bars, they would be inappropriate for our tests because the high conductivity of copper (~400 Wm^−1^K^−1^) would obscure the signal from our very insulating spacers. Thus, we modified the technique, replacing the copper rods with less conducting polyimide rods (~1 Wm^−1^K^−1^). In contrast to copper, polyimide also has a high thermal emissivity (close to unity), allowing us to read out the temperature distribution across the meter bars with a thermal infrared camera instead of embedded thermocouples or other more invasive sensors.

Figure S4 shows the experimental vacuum chamber setup for thermal conductance measurement. Full details of the experimental process are provided in Supplemental Section S5. A thermionic converter was experimentally simulated by separating two metal-coated (typically ~100 nm tungsten) silicon chips with a spacer sample and heating the bottom polyimide rod. All temperature measurements were taken under vacuum with an external thermal infrared camera (example image shown in Fig. 4b). Though we were able to easily obtain higher resolution temperature distributions with the thermal camera (compared to discrete embedded temperature sensors), we still needed to average ~1000 image frames to reduce the noise and obtain confident fits from the temperature data. We gradually applied a compressive force to the sandwich, allowing us to determine the thermal conductance as a function of compressive pressure and the capacitively measured gap distance.

## Supplementary information

Supplemental Material

## References

[1] Hatsopoulos, G. N. *Thermionic energy conversion*. (MIT Press, 1979).

[2] Schwede JW (2010). Photon-enhanced thermionic emission for solar concentrator systems. Nat. Mater..

[3] Lee J-H, Bargatin I, Melosh NA, Howe RT (2012). Optimal emitter-collector gap for thermionic energy converters. Appl. Phys. Lett..

[4] Segev G, Weisman D, Rosenwaks Y, Kribus A (2015). Negative space charge effects in photon-enhanced thermionic emission solar converters. Appl. Phys. Lett..

[5] Narayanaswamy A, Chen G (2003). Surface modes for near field thermophotovoltaics. Appl. Phys. Lett..

[6] Fiorino A (2018). Nanogap near-field thermophotovoltaics. Nat. Nanotechnol..

[7] Lim M (2018). Optimization of a near-field thermophotovoltaic system operating at low temperature and large vacuum gap. J. Quant. Spectrosc. Radiat. Transf..

[8] Zhao B (2017). High-performance near-field thermophotovoltaics for waste heat recovery. Nano Energy.

[9] Go, D. B. et al. Thermionic energy conversion in the twenty-first century: advances and opportunities for space and terrestrial applications. *Front. Mech. Eng*. **3**, (2017).

[10] Cahill DG, Watson SK, Pohl RO (1992). Lower limit to the thermal conductivity of disordered crystals. Phys. Rev. B.

[11] Gorham CS, Gaskins JT, Parsons GN, Losego MD, Hopkins PE (2014). Density dependence of the room temperature thermal conductivity of atomic layer deposition-grown amorphous alumina (Al_2_O3). Appl. Phys. Lett..

[12] Costescu RM, Cahill DG, Fabreguette FH, Sechrist ZA, George SM (2004). Ultra-low thermal conductivity in W/Al_2_O_3_ nanolaminates. Science.

[13] Goto M (2018). Ultra-low thermal conductivity of high-interface density Si/Ge amorphous multilayers. Appl. Phys. Express.

[14] Alvarez-Quintana J, Peralba-Garcia L, Lábár JL, Rodríguez-Viejo J (2009). Ultra-low thermal conductivity in nanoscale layered oxides. J. Heat. Transf..

[15] Ali S (2016). Thermal conductivity of amorphous Al_2_O_3_/TiO_2_ nanolaminates deposited by atomic layer deposition. Nanotechnology.

[16] Döring F, Major A, Eberl C, Krebs H-U (2016). Minimized thermal conductivity in highly stable thermal barrier W/ZrO_2_ multilayers. Appl. Phys. A.

[17] Hrubesh LW, Pekala RW (1994). Thermal properties of organic and inorganic aerogels. J. Mater. Res..

[18] Koebel M, Rigacci A, Achard P (2012). Aerogel-based thermal superinsulation: an overview. J. Sol.-Gel Sci. Technol..

[19] Zircar Ceramics. Microporous Insulation Type MICROSIL.

[20] Aerogels Handbook. (Springer-Verlag, 2011).

[21] Home | Airloy Ultramaterials: Strong Aerogels from Aerogel Technologies. Available at: http://www.airloy.com/category/home/. (Accessed: 2nd July 2018).

[22] Aspen Aerogels. Aspen Aerogels Pyrogel XTE Spec Sheet.

[23] Weinstein LA (2018). A hybrid electric and thermal solar receiver. Joule.

[24] Jr RGR (2015). Quantifying MLI thermal conduction in cryogenic applications from experimental data. IOP Conf. Ser.: Mater. Sci. Eng..

[25] Fesmire JE, Johnson WL (2018). Cylindrical cryogenic calorimeter testing of six types of multilayer insulation systems. Cryogenics.

[26] Kutzner K, Schmidt F, Wietzke I (1973). Radiative and conductive heat transmission through superinsulations — experimental results for aluminium coated plastic foils. Cryogenics.

[27] Reiss H (2004). A coupled numerical analysis of shield temperatures, heat losses and residual gas pressures in an evacuated super-insulation using thermal and fluid networks: Part I: Stationary conditions. Cryogenics.

[28] Lee, J. H. et al. Encapsulated thermionic energy converter with stiffened suspension. in Proceedings of Solid-State Sensors, Actuators, and Microsystems Workshop 493–496 (Transducer Research Foundation, 2012).

[29] Lee JH (2014). Microfabricated thermally isolated low work-function emitter. J. Micro. Syst..

[30] Fitzpatrick, G. O., Koester, J. K., Chang, J., Britt, E. J. & McVey, J. B. Close-spaced thermionic converters with active spacing control and heat-pipe isothermal emitters. in Energy Conversion Engineering Conference, 1996. IECEC 96, Proceedings of the 31st Intersociety 2, 920–927 vol. 2 (1996).

[31] Beggs JE (1963). Vacuum thermionic energy converter. Adv. Eng. Conv..

[32] King, D. B., Zavadil, K. R., Jennison, D. R., Battaile, C. C. & Marshall, A. C. *Low Work Function Material Development for the Microminiature Thermionic Converter*. (Sandia National Laboratories, 2004).

[33] Littau KA (2013). Microbead-separated thermionic energy converter with enhanced emission current. Phys. Chem. Chem. Phys..

[34] Belbachir, R. Y., An, Z. & Ono, T. Thermal investigation of a micro-gap thermionic power generator. *J. Micromech. Microeng*. 085009 (2014).

[35] DiMatteo R (2004). Micron‐gap ThermoPhotoVoltaics (MTPV). AIP Conf. Proc..

[36] Belbachir RY, An Z, Ono T (2016). Impact of thermal contact resistances on micro-gap heat losses for microthermionic power generators. Micro. Technol..

[37] Ito K, Miura A, Iizuka H, Toshiyoshi H (2015). Parallel-plate submicron gap formed by micromachined low-density pillars for near-field radiative heat transfer. Appl. Phys. Lett..

[38] Cappella A (2013). High temperature thermal conductivity of amorphous Al_2_O_3_ thin films grown by low temperature ALD. Adv. Eng. Mater..

[39] Davami K (2015). Ultralight shape-recovering plate mechanical metamaterials. Nat. Commun..

[40] Scherer GW, Smith DM, Qiu X, Anderson JM (1995). Compression of aerogels. J. Non-Cryst. Solids.

[41] Woignier T, Phalippou J (1988). Mechanical strength of silica aerogels. J. Non-Cryst. Solids.

[42] Parmenter KE, Milstein F (1998). Mechanical properties of silica aerogels. J. Non-Cryst. Solids.

[43] Madhusudana, C. V. *Thermal Contact Conductance*. (Springer-Verlag, 1996).

[44] Bowden, F. P. & Tabor, D. *The Friction and Lubrication of Solids*. (Oxford University Press, 2001).

[45] Llewellyn-Jones, F. *The physics of electrical contacts*. (Clarendon Press, 1957).

[46] Holm, R. *Electric Contacts: Theory and Application*. (Springer-Verlag, 1967).

[47] Carslaw, H. S. & Jaeger, J. C. *Conduction of Heat in Solids*. (Oxford University Press, 1986).

[48] Gwyddion – Free SPM data analysis software. Available at: http://gwyddion.net/. (Accessed: 29th August 2018).

[49] ASTM International. ASTM D5470-17 Standard Test Method for Thermal Transmission Properties of Thermally Conductive Electrical Insulation Materials. (ASTM International, 2017).

[50] Smith, A. N., Jankowski, N., Boteler, L. & Meyer, C. Interfacial Resistance Measurement of High Performance Thermal Interface Materials. in Proceedings of ASME 2013 Heat Transfer Summer Conference HT2013, V003T10A003 (ASME, 2013).

[51] Jarrett, R. N., Merritt, C. K., Ross, J. P. & Hisert, J. Comparison of Test Methods for High Performance Thermal Interface Materials. in 2007 PROCEEDINGS, Twenty Third IEEE Semiconductor Thermal Measurement and Management Symposium 07CH37872, (IEEE, 2007).

[52] Kempers R, Kolodner P, Lyons A, Robinson AJ (2009). A high-precision apparatus for the characterization of thermal interface materials. Rev. Sci. Instrum..

[53] Zhao, Y., Chu, R.-S. & Majumdar, A. Transient thermo-reflectance method for characterization of thermal interface material based on carbon nanotube array. in Proceedings of the ASME 2009 Second International Conference on Micro/Nanoscale Heat and Mass Transfer 2, 435–442 (ASME, 2009).

[54] Hu XJ, Padilla AA, Xu J, Fisher TS, Goodson KE (2005). 3-omega measurements of vertically oriented carbon nanotubes on silicon. J. Heat. Transf..

