# Supplementary material for: Micron-gap spacers with ultrahigh thermal resistance and mechanical robustness for direct energy conversion
Source: Microsyst Nanoeng. 2019 Jul 15;5:31. doi: 10.1038/s41378-019-0071-4 (PMC6799816; doi:10.1038/s41378-019-0071-4)
Supplement: Supplementary file 1 — Supplemental Material [file 41378_2019_71_MOESM1_ESM.docx]

# Micron-gap spacers with high thermal resistance and mechanical robustness for direct energy conversion

*S. M. Nicaise^1*^, C. Lin^1*^, M. Azadi^1^, T. Bozorg-Grayeli^3^, P. Adebayo-Ige^2^, D.E. Lilley^1^, Y. Pfitzer^1^, W. Cha^1^, K. Van Houten^4^, N. Melosh^3^, R. T. Howe^5^, J. W. Schwede^4^, I. Bargatin^1^*

^1^Mechanical Engineering and Applied Mechanics, University of Pennsylvania, Philadelphia, PA, USA

^2^Chemical and Biomolecular Engineering, University of Pennsylvania, Philadelphia, PA, USA

^3^Materials Science & Engineering, Stanford University, Stanford, CA, USA

^4^Spark Thermionics, Berkeley, CA, USA

^5^Electrical Engineering, Stanford University, Stanford, CA, USA

* These authors contributed equally to this work.

Supplemental Section S1: COMSOL Finite Element Simulations

Numerical simulations were carried out to model the small-deformation tensile behavior, including geometric nonlinearity. During the simulations, the spacer plate height and plate thickness were set as 2 $\mu m$ and 800 nm, respectively, and the 3D models were made of alumina with isotropic linear elastic properties (assuming Young’s modulus as 130 GPa and Poisson’s ratio as 0.22 (ref. 1,2). The in-plane simulations were performed by applying $0.1\%$ tensile strains ($\varepsilon_{t}$) at all the spacer array boundaries along both of the in-plane *x* and *y* directions. We also used COMSOL to model the compressive stiffness of the spacer designs, including geometric nonlinearity. The bottom plane of a spacer was fixed and a compression pressure of 1 atm was applied at the top plane in the out-of-plane *z* direction. Compared to straight hexagonal designs, both wavy and expanding designs showed reduced stiffness for biaxial in-plane extension and increased robustness, as indicated by the reduced maximum principal and von Mises stresses (Table S1).

*Table S1: Top-down schematics of the different spacer designs. The four on the right were designed to provide in-plane compliance. The table provides in-plane tensions and out-of-plane compression mechanical results for COMSOL finite element simulations.* $\bar{W}$*: average elastic strain energy density;* $\sigma_{pmax}$*: maximum principal stress;* $\sigma_{vmax}$*: maximum von Mises stress;* $E_{ceff}$*: out-of-plane effective compressive modulus;*$u_{z}$*: out-of-plane displacement;* $\sigma_{ceff}$*: out-of-plane effective compressive stress;* $\sigma_{pmax}$*: maximum principal stress;* $\sigma_{vmax}$*: maximum von Mises stress;* $\bar{W}$*: average elastic strain energy density).*


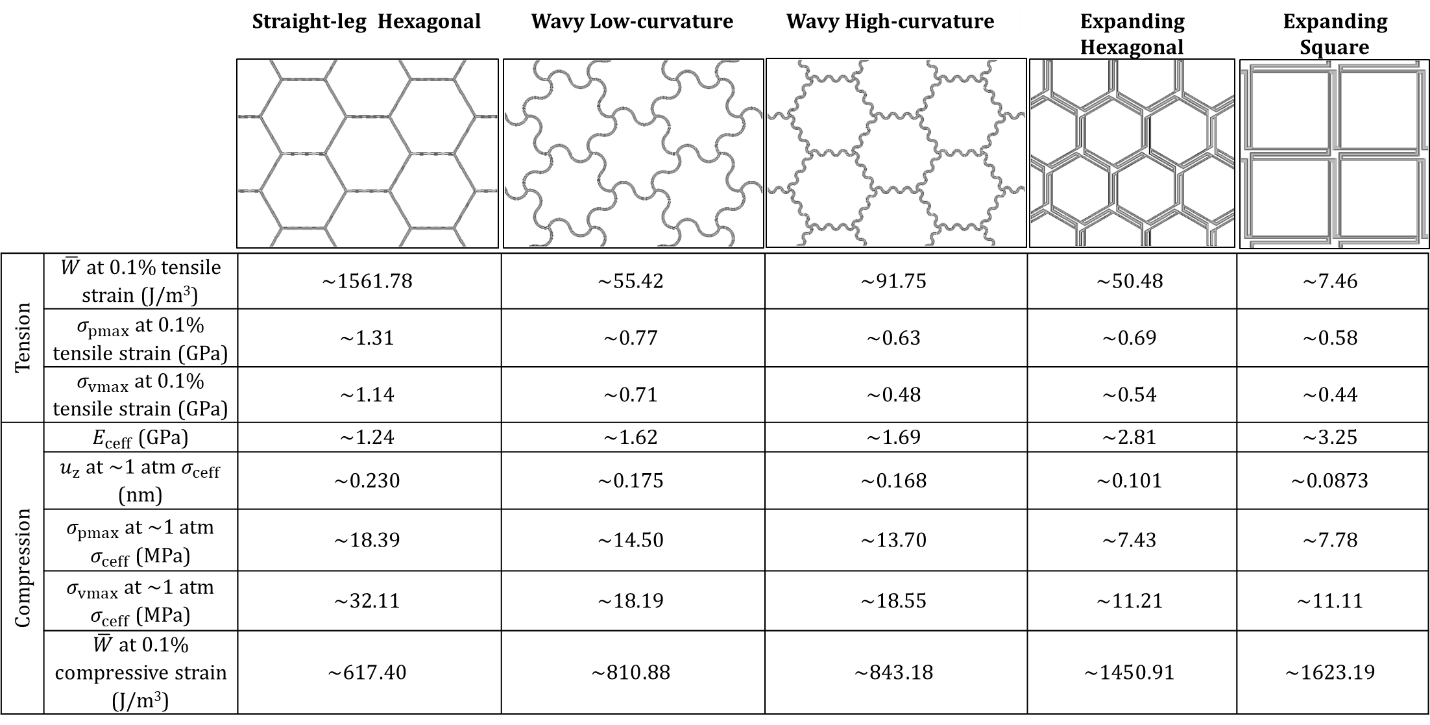


Supplemental Section S2: Fabrication

Figure 2 in the main text shows the fabrication process of the spacer device. Figure 1c in the main text shows a photograph of a centimeter-sized released spacer sample sitting on a molybdenum mirror, along with top down optical microscope images in Figure S1. These images clearly highlight the ribs, openings and inter-electrode gap distance provided by the samples. On all of the samples, the U-beam ribs had flanges on the bottom side as a result of the lithography process. We minimized the flange size, and they were generally 1-5 μm in width. While the flanges likely contributed to the thermal characteristics of the spacers, this study did not have an adequate way to determine the contribution of the flanges in thermal experiments or theoretical models.


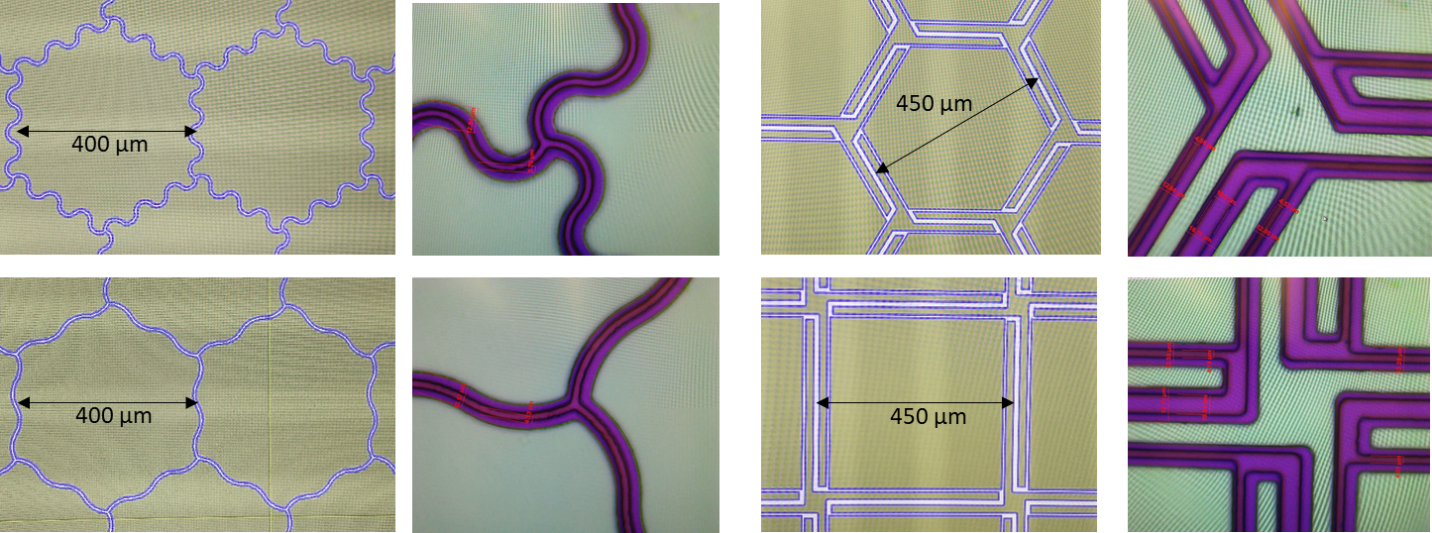


*Figure S1: Top-down as-designed dimensions of the different spacers. These images are examples of the spacer mask design and final alumina before the isotropic etch of the silicon mold.*

The spacer height and thickness can be in-principle be reduced down to ~100 nm and ~10 nm, respectively, using the same fabrication techniques. The main practical challenge with such small height and thickness is the difficulty in transferring the fabricated spacer from the mold substrate to the experimental substrate. Although we successfully fabricated and released a spacer with thicknesses as low as 75 nm, we did not test them because all spacers with centimeter-scale diameter and thicknesses < 200 nm were very floppy and difficult to handle. The height in our experiments was reduced down to below 3 μm with the U-beam ribs but reducing the spacer height below 2 μm was challenging because of greatly increased chance of electrical and thermal shorts. Transfer was possible with these samples, albeit difficult by hand. Based on our experience, the wafer flatness and roughness become much more important at the sub-1-micron gap length scale and non-uniformities at the edges of the electrodes often prevent the spacer from maintaining a very small gap. In our study, we did not work to perfect the electrode flatness and lack of edge-roughness, though it would be necessary for a practical device. In principle, we expect that our spacer design can be scaled down to *d* ≤ 1 μm and *t* ≤ 100 nm in the future if the transfer is optimized and electrodes are perfectly flat and clean.

Supplemental Section S3: Mechanical Compression Characterization

Spacer samples were placed on 0.25” thick glass which was on top of a custom-built test stand. On top of the spacer was placed a tungsten-coated silicon chip, a few millimeters by a few millimeters, and a chip of a microscope slide (not pictured) in order to distribute the pressure and prevent bowing of the silicon chip. Alternatively, we used a polished, highly-planar molybdenum mirror, 0.5” in diameter, in order to simulate the electrode. A ball tip indenter, with a tip of 1.5 mm radius, was attached to the load cell of the Instron in order to ensure self-alignment of the glass, spacer and top electrode. Compression was applied until complete and catastrophic failure of the spacer, which was observed at 387-3870 kPa. In most experiments, it was obvious that the pressure was uniformly distributed because we did not see any difference in the fracture of the spacer across the sample area. Also important, the spacer failed catastrophically, as obvious from the microscope images and abrupt changes in the force-displacement curve, instead of slowly yielding in one part of the sample before the other. Figure 3c in the main text provides an example stress-displacement curve, showing a nearly linear increase in pressure until failure above 2000 kPa. Similar curves were obtained for most of the samples, in which the measured stress decreased significantly at the same time as we observed failure in the video microscope. Figure 3d in the main text shows an example of a stress-strain curve for a ~800 nm thick, ~4.2 μm tall expanding hexagonal spacer in which the strain was measured precisely with the capacitive setup. From this graph, the linearly-fitted slope gives us the effective compressive modulus of the spacer.


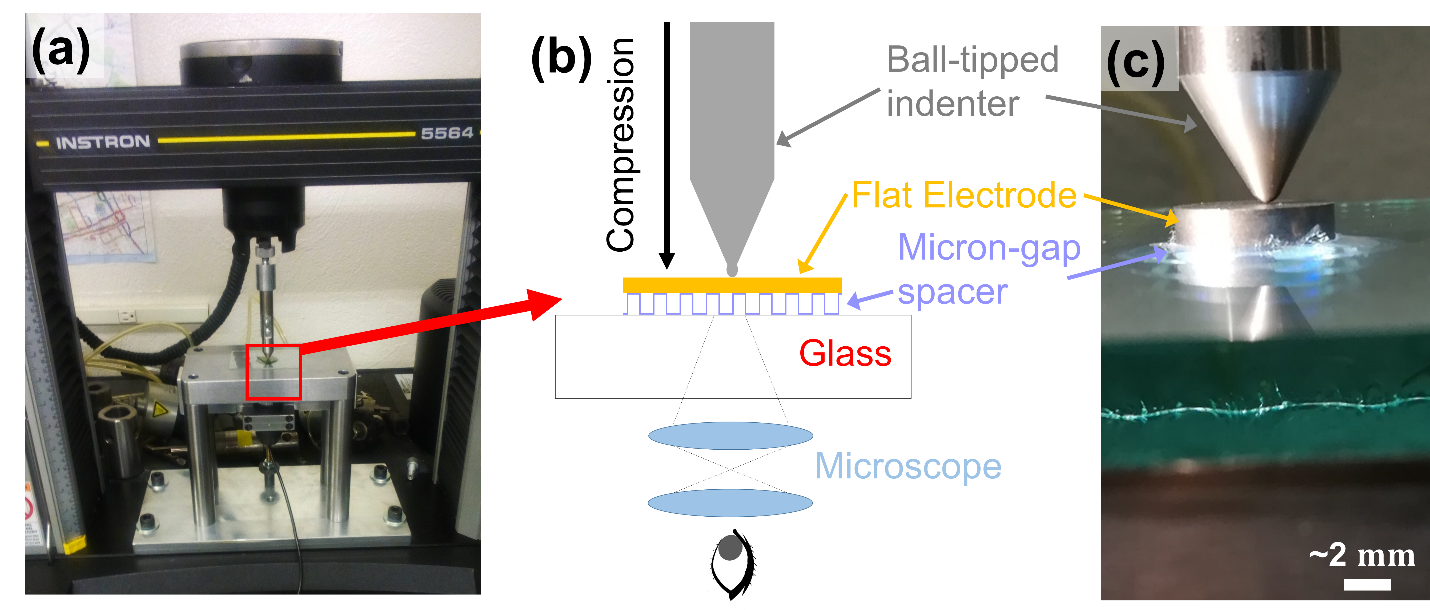


*Figure S2: Compression setup and images of spacers. a-c) Photos and a schematic of the tool setup in which an Instron materials tester was used to apply compressive force onto a stack of glass-spacer-electrode.*

*Table S2: Failure Strength of various spacer samples*

|  | Dimensions | Failure Pressure (kPa) |
| --- | --- | --- |
| Expanding Square | 4.25 μm tall, 800 nm thick | ~1534, ~3700 |
| Wavy Low-Curvature | 4.25 μm tall, 800 nm thick | ~2870, ~3870 |
| Expanding Hexagonal (1) | 4.25 μm tall, 200 nm thick | ~387 |
| Expanding Hexagonal (2) | 4.25 μm tall, 800 nm thick | ~2034 |

Supplemental Section S4: Measuring the Micron-scale Gap

A sinusoidal voltage source $V_{\mathrm{source}}$, 100 mV root-mean squared (RMS) and frequency $\omega$ was applied to a reference resistor $R_{\mathrm{ref}}$ of 213700 Ω in series with the to-be-measured device capacitor (stack of electrode-spacer-electrode). The RMS voltage across the capacitor $V_{C}$ was measured by a Zurich Instruments HF2LI lock-in amplifier which was filtering at the sourced frequencies of 5, 10 and 20 kHz. Based on series circuit voltage division, $\frac{V_{C}}{V_{\mathrm{source}}}=\frac{1/j\omega C}{R_{\mathrm{ref}}+1/j\omega C}$. Also, according to the parallel plate capacitor formula, $C=\frac{\varepsilon_{r}\varepsilon_{0}A}{d}$, where $\varepsilon_{r}$ is the relative permittivity ($\sim1$ since the space between the two silicon pieces was mostly in vacuum), $\varepsilon_{0}$ is the vacuum permittivity ($\sim8.854\times{10}^{-12}F\cdot m^{-1}$), $A$ is the effective capacitor area which we measured with calipers, and $d$ is the to be calculated gap distance. As a result, $d=\varepsilon_{0}A\sqrt{\frac{\omega^{2}R_{\mathrm{ref}}^{2}V_{C}^{2}}{V_{\mathrm{source}}^{2}-V_{C}^{2}}}$. The uncertainty in the gap measurement is estimated to be 0.5 μm based on the precision of $V_{C}$ and error in *A* and *R*_ref_.


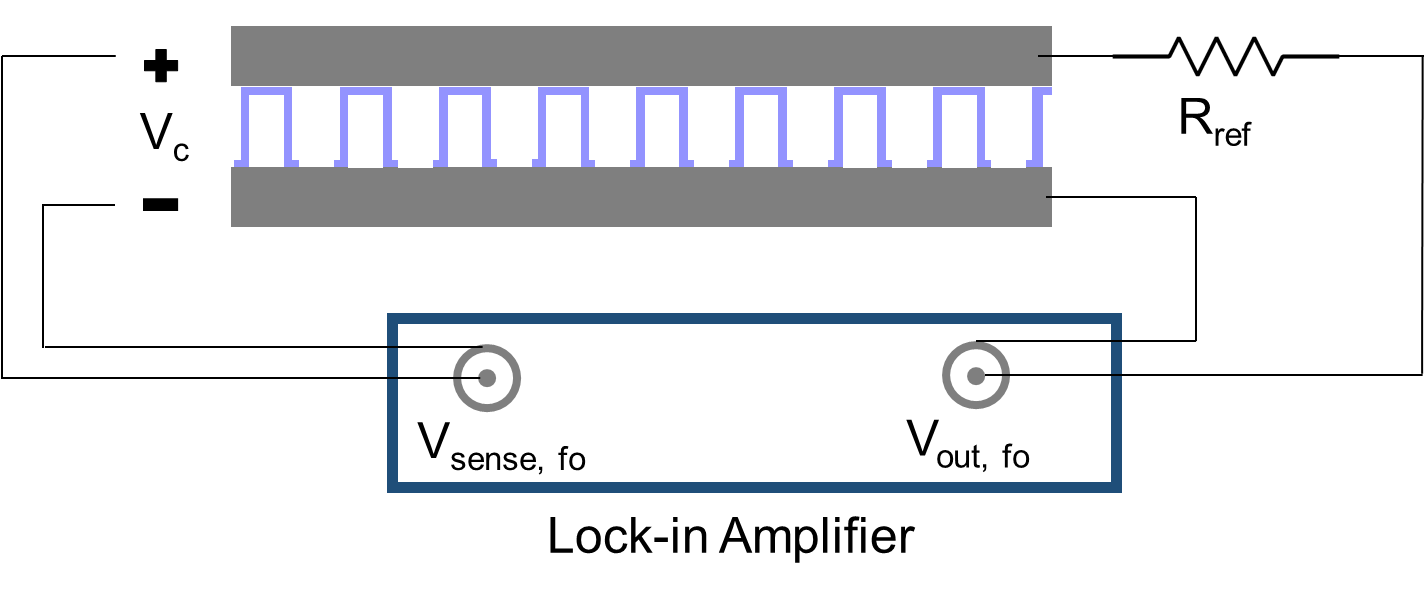


*Figure S3: Schematic of the electrical setup to measure the capacitance and therefore gap distance between two planar electrodes with a spacer in between. Right side: the alternating-current supply voltage was split between a reference resistor and the device capacitor. Left side: the complex voltage across the device capacitor was measured with the lock-in amplifier at the specific output frequency.*

Supplemental Section S5: Thermal Characterization

Silicon wafers were coated on the polished side with a thin film of tungsten (or, in a few cases, gold) (~100 nm via sputter deposition). Small chips of roughly 6×15 mm that were cleaved from the wafers simulated the device electrodes. We chose DuPont^TM^ Vespel^®^ SP-1 polyimide cylindrical rods as the meter bar material instead of standard copper because the lower thermal conductivity of polyimide, $\sim1$ W m^-1^ K^-1^ vs. $\sim400$ W m^-1^ K^-1^, simplified the measurements of the very low thermal conductances of our spacers. Thermally conductive tape (Blue Cell BCP, conductivity specification of 1 W m^-1^ K^-1^, measured thickness ~100 μm) acted as the thermal interface material between the polyimide rods and the silicon electrodes. A spacer sample was placed on the bottom electrode and then the top assembly (polyimide rode with taped silicon electrode) slowly brought into contact (light pressure) to form the interface stack. It should be noted that the top surface of the bottom polyimide rod was slightly rounded (0.0926 mm^-1^ curvature based on caliper measurement), which contributed to the planar self-alignment between the two electrodes. The vacuum chamber was then evacuated with a roughing and turbo pump for 3-24 hours to a pressure below 2×10^-6^ T. We could apply variable force to the interface sandwich, along the axial/out-of-plane direction. The net force was measured with a Wheatstone bridge-based Honeywell FSG piezoresistive force sensor underneath the bottom polyimide rod by subtracting the null output from just the bottom half of the device stack. The net applied pressure was then determined based on the measured overlap area of the two electrodes.


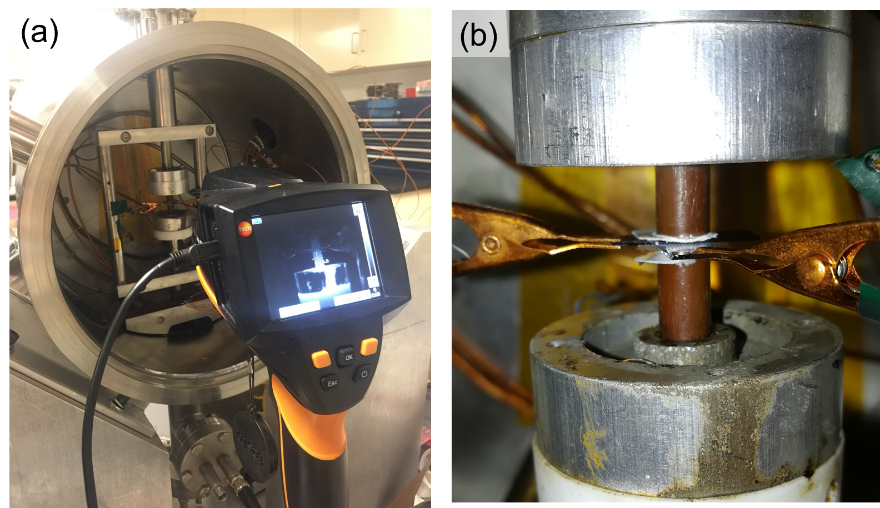


Figure S4: Images of the thermal characterization setup. (a) The open vacuum chamber with the thermal imaging camera (in the actual tests, the vacuum chamber was closed and the camera imaged through the view port). (b) Close up image of stacked electrodes and spacer with alligator clips for sensing the capacitance of the stack. The heater is at the bottom and conducted through the brown polyimide rods.

The temperature of the bottom electrode $T_{\mathrm{cathode}}$ reached to $80$-200 $℃$ and, therefore, we assumed the thermal radiation heat flow increase linearly with $T_{\mathrm{cathode}}$. Using a Testo 875i thermal imager (thermal sensitivity at 30 $℃$: < 50 mK), we measured the temperature drop across the spacer device ${\Delta T}_{\mathrm{drop}}$ and the temperature profile along the polyimide rods. One example thermal infrared image is shown in Figure 4b in the main text. The foundational equations for the model are given in the main text. We calculated $\frac{dT\left( y \right)}{dy}|_{\mathrm{interface}}$ by fitting the measured temperature distribution to the fin-structure heat transfer formula $T\left( y \right)-T_{\infty}=C_{1}e^{mx}+C_{2}e^{-mx}$ with $m^{2}=\frac{hP_{c}}{k_{\mathrm{polyimide}}A_{c}}$, where $T_{\infty}$ is the ambient temperature, $m$, $C_{1}$ and $C_{2}$ are three constants, $h$ is the heat transfer coefficient of the sidewalls of the meter bars, $P_{c}$ is the polyimide cross section perimeter, $k_{\mathrm{polyimide}}$ is the polyimide thermal conductivity (calibrated as $\sim1$ W m^-1^ K^-1^ by using a single long polyimide rod to extract $k_{\mathrm{polyimide}}$ from the fitted $m$), and $A_{c}$ is the polyimide rod cross-sectional area. The calculated value for $C_{\mathrm{thermal}}$ can then be inverted to give $R_{\mathrm{measured}}$ as the thermal resistance that prevents heat flow between the electrodes. Figure 4c, in the main text, shows one example where we averaged 1000 thermal images and fitted the above fin-structure transfer formula to each rod with the constant flux assumption. The *x* axis represents the length along the rod, and *y* axis represents the difference between the polyimide rod temperature and ambient temperature at each pixel along the rods.

Supplemental Section S6: Asperity-based Contact Resistance and Sparse Contact Points

Given the macroscopic size of our spacer samples, we expect that more than a single asperity was in contact, and thus we can model the contact with the multiple spot contact conductance $h_{s}$. We also assume that we can disregard thermal radiation and convection in our vacuum setup^3^. In this model, the conductance between spots on side 1 and side 2 is given by $h_{s}=2na_{m}k_{\mathrm{eff}}/F$, where $k_{\mathrm{eff}}=2k_{1}k_{2}/(k_{1}+k_{2})$ represents the harmonic mean of the thermal conductivities, $F$ represents the constriction alleviation factor (typically, approximately equal to one), $n$ is the number of contact spots at the interface, and $a_{m}$ is the average radius of the contact spots. Previous reports provided surface and deformation analyses in order to estimate $a_{m}$ and $n$ ^4–8^. Based on the report of Mikic et al., we can assume that:

$h_{s}=1.55\frac{k_{\mathrm{eff}}s_{\mathrm{eff}}}{\sigma_{\mathrm{eff}}}{(\frac{P\sqrt{2}}{E^{'}s_{\mathrm{eff}}})}^{0.94}$ where $P$ is the applied mechanical pressure between the contact surfaces, $H$ is the microhardness of the softer material, $E^{'}=\left[ \frac{1-\nu_{1}^{2}}{E_{1}}+\frac{1-\nu_{2}^{2}}{E_{2}} \right]^{-1}$ is the reduced elastic modulus of solids 1 and 2, $\sigma$ is the roughness of the equivalent surface, and $s_{\mathrm{eff}}$ is the slope of the equivalent surface, assuming that our surfaces are locally rough but globally flat and deform elastically^9^.

The important parameters were calculated as follows. RMS roughness $\sigma=\sqrt{\frac{1}{n}\sum_{i=1}^{n} z_{i}^{2}}$, where $z_{i}$ represents the vertical distance from the mean along a line in the *x* direction), mean grain sizes $\bar{D}$, and the average absolute slope estimated as$s\approx8\times\sigma/\bar{D}$ (strictly defined as $s=\frac{1}{N}\sum_{i=1}^{N} \left| \Delta_{i} \right|$, $\Delta_{i}=\frac{1}{60}\times\frac{z_{i+3}-9z_{i+2}+45z_{i+1}-45z_{i-1}+9z_{i-2}-z_{i-3}}{\Delta x}$ based on ASME B46.1 (ref. 10)). Our measurements are listed in Table S3 based on the atomic force micrographs in Figure S4. Thus, for either the top or bottom contact surface, the effective RMS surface roughness can be calculated as $\sigma_{\mathrm{eff}}=\sqrt{\sigma_{\mathrm{tungsten}}^{2}+\sigma_{\mathrm{alumina}}^{2}}$ and the effective slope can be calculated as $s_{\mathrm{eff}}=\sqrt{s_{\mathrm{tungsten}}^{2}+s_{\mathrm{alumina}}^{2}}$ ^3^, which are also shown in Table S3. The Young’s moduli, Poisson’s ratios, thermal conductivities of tungsten and ALD alumina are $E_{\mathrm{tungsten}}=411$ GPa, $\nu_{\mathrm{tungsten}}=0.28,\mathrm{and}k_{\mathrm{tungsten}}=173$ W m^-1^ K^-1^, and $E_{\mathrm{alumina}}=130$ GPa, $\nu_{\mathrm{alumina}}=0.22, and k_{\mathrm{alumina}}=\sim2$ W m^-1^ K^-1^ (ref. 1,2,11). Thus the reduced elastic modulus is $E^{'}=\left[ \frac{1-\nu_{\mathrm{tungsten}}^{2}}{E_{\mathrm{tungsten}}}+\frac{1-\nu_{\mathrm{alumina}}^{2}}{E_{\mathrm{alumina}}} \right]^{-1}\approx1.046\times{10}^{11}$ Pa and the effective thermal conductivity is $k_{\mathrm{eff}}=\frac{2k_{\mathrm{tungsten}}k_{\mathrm{alumina}}}{k_{\mathrm{tungsten}}+k_{\mathrm{alumina}}}\approx3.954$ W m^-1^ K^-1^.


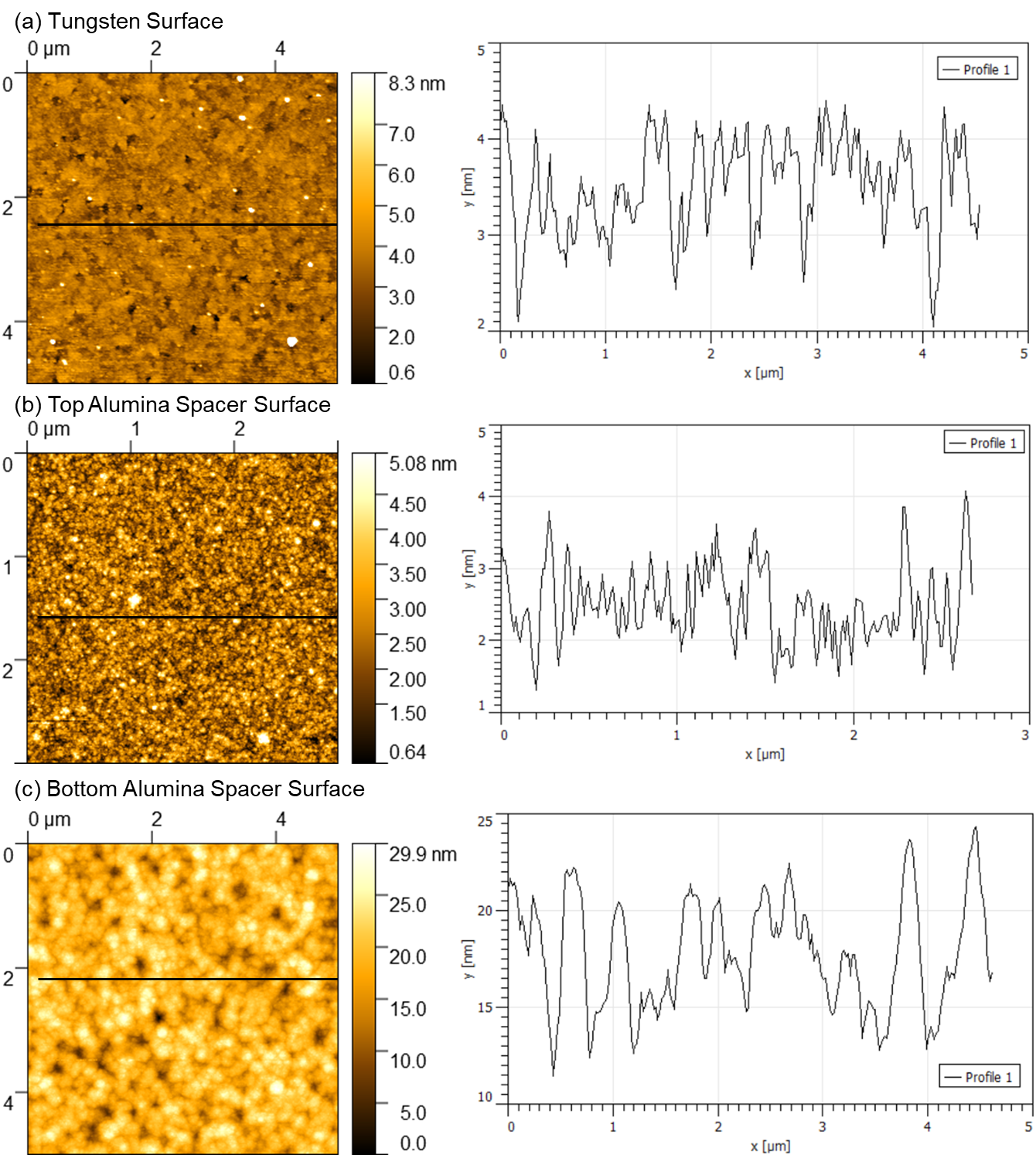


Figure S5: AFM topographic images and line scans for the different surfaces in the device sandwich. (a) Tungsten surface on silicon substrate, (b) top alumina spacer surface and (c) bottom alumina spacer surface. The line scans show examples of the surface roughness.

Table S3: Estimated RMS roughnesses, mean grain sizes and average absolute slopes.

|  | | Tungsten | Alumina | Effective Tungsten/Alumina Surface |
| --- | --- | --- | --- | --- |
| RMS Roughness $\sigma$ (nm) | Top | $\sim0.720$ | $\sim0.672$ | $\sim0.985$ |
|  | Bottom | $\sim0.720$ | $\sim3.432$ | $\sim3.507$ |
| Mean Grain Size $\bar{D}$ (nm) | Top | $\sim50$ | $\sim31.75$ | N/A |
|  | Bottom | $\sim50$ | $\sim127$ |  |
| Average Absolute Slope $s$ | Top | $\sim0.115$ | $\sim0.169$ | $\sim0.204$ |
|  | Bottom | $\sim0.115$ | $\sim0.216$ | $\sim0.245$ |

We fitted the experimentally measured thermal resistance $R_{\mathrm{measured}}$ as a function of the apparent applied pressure $P_{\mathrm{app}}$, shown in Figure 5a in the main text, based on the formula

$$R_{\mathrm{measured}}\approx R_{\mathrm{standoffs}}+R_{\mathrm{tcontact}}+R_{\mathrm{bcontact}}=R_{\mathrm{standoffs}}+\frac{1}{h_{\mathrm{ts}}}+\frac{1}{h_{\mathrm{bs}}}$$

$$\approx R_{\mathrm{spacer}}+\left\{ \left[ \frac{\sigma_{\mathrm{efft}}}{{(s_{\mathrm{efft}} )}^{0.06}}+\frac{\sigma_{\mathrm{effb}}}{{(s_{\mathrm{effb}} )}^{0.06}} \right]\frac{{{(E}^{'})}^{0.94}}{1.55\left( \sqrt{2} \right)^{0.94}k_{\mathrm{eff}}} \right\}\times\frac{1}{P_{\mathrm{app}}^{0.94}}$$

$=R_{\mathrm{spacer}}+C\times\frac{1}{P_{\mathrm{app}}^{0.94}}$

where *s*_eff_ and *σ*_eff_ are different for the top and bottom sides of the spacer and the effective *E* and *k* are assumed to be the same for the top and bottom.

We extracted the fitting parameters $R_{\mathrm{spacer}}$ and $C$ for data sets of spacers with varying gap distance $d$ and plate thickness $t$. The fitted $R_{\mathrm{spacer}}$ varied from $\sim3.76\times{10}^{-2}-6.51\times{10}^{-2}$ cm^2^ K mW^-1^ for different spacer array samples. Figure S6a and S6b shows the dependence of $R_{\mathrm{spacer}}$ on gap distance (at a constant thickness of 800 nm) and thickness (at a constant gap distance of 4-5 μm), respectively. A linear fit of the data in Figure S6a approximates an effective thermal conductivity of $\sim5$ mW m^-1^ K^-1^ if we consider the spacer arrays to be analogous to an effective bulk material. As a comparison, some of the most insulating aerogels have thermal conductivities in the range of 10-30 mW m^-1^ K^-1^. We note here that the conductivity of the spacer is based strictly on the measurements of *R*_spacer_ and that the contact resistance or cross-sectional area of aerogels may differ from the spacer. Also, these measurements are in vacuum, where the performance of the spacer is ideal; aerogels would most certainly be less conducting than the spacers if measured in an air environment. Multi-layer insulators tend to be at least as insulating as our spacer, and in most cases up to 10 times more insulating, though multi-layer assemblies tend to be much thicker and cannot sustain applied pressures on the order of 100 kPa. (see the discussion of aerogels and multi-layer assemblies in the main text.)


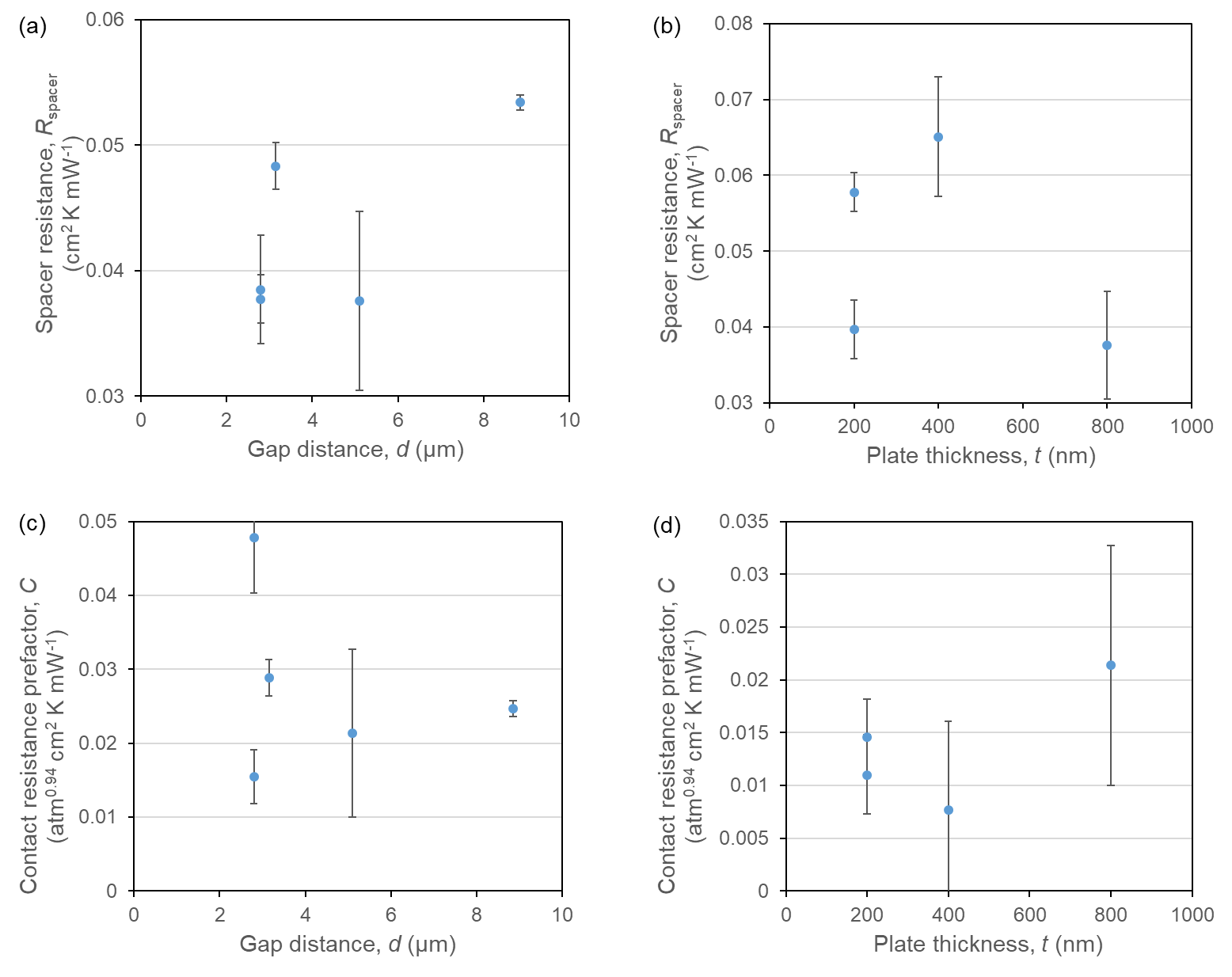


Figure S6: Results of the contact resistance spacer resistance fitting

The extracted value of $C$ is plotted in Figure S6c vs. gap distance (at a constant thickness of 800 nm) and thickness (at a constant gap distance of 4-5 μm), respectively, and varied from ${\sim7.73\times10}^{-3}$ to ${\sim4.78\times10}^{-2}$ atm^0.94^ cm^2^ K mW^-1^. In order to compare these values to a theoretical expectation of $C$ between the tungsten coating surface and the ALD alumina spacer surface, we characterized the profile of surfaces with atomic-force microscopy, detailed in in Figure S4. The theoretical $C$ was computed as $\left[ \frac{\sigma_{\mathrm{efft}}}{{(s_{\mathrm{efft}} )}^{0.06}}+\frac{\sigma_{\mathrm{effb}}}{{(s_{\mathrm{effb}} )}^{0.06}} \right]\frac{{{(E}^{'})}^{0.94}}{1.55{(\sqrt{2})}^{0.94}k_{\mathrm{eff}}}\approx13.172$ Pa^0.94^ cm^2^ K W^-1^$\approx2.596\times{10}^{-3}$ atm^0.94^ cm^2^ K mW^-1^, which is a factor of 3-18 smaller than the experimental values from the fitted curves. This discrepancy is reasonable considering the variability of our experimental setup, the small size of the samples, and mismatch between the assumed planar surfaces of the model and the non-uniform design of our spacer arrays. Other possible causes for this discrepancy include macroscopic bowing in the spacer, misalignment of the electrodes, and the assumed value of $k_{\mathrm{alumina}}$=$2$ W m^-1^ K^-1^, which is based on literature reports and may vary for our deposited films. Nonetheless, the higher than expected contact resistance could provide a beneficial enhancement in thermal insulation for thermionic converters or other devices when operating at lower contact pressures.

For the calculation of density of the contact spots in the main text (Figure 5d), our model assumed that one of the single contact point has a width similar to the plate thickness and only slightly larger than the grain sizes of the alumina, thus on the order of 100s-of-nanometers.

As explained below, we determined that the sparse contact points would necessarily be co-located opposite each other on the top and bottom of the rib walls when as little as 100 kPa of pressure was applied. In order to determine this, we considered the rib wall to be a tall, thin and long beam that was simply supported underneath at two ends and had a force applied in the middle, similar to three point bending. The force was based on the sparse nonuniform contact model presented in the main text, in which the absolute force was calculated by dividing 100 kPa of pressure over the total number contact points, which was a function of the distance between points. The wall also had flanges, 1 μm wide on the top and 5 μm wide on the bottom, similar to the fabricated ribs. We assumed that the sides of the beam had asperities with height similar to the roughness measured from the AFM scans (above). We then calculated the displacement that would occur in the middle of the beam, at the point of the applied force, for different distances between adjacent points. Table S4 shows the calculated displacements with the following assumptions: expanding hexagonal design with a unit cell area of 0.0000175 m^2^, spacer height of 4.5 μm, and ALD alumina with a Young’s modulus *E* of 130 GPa and shear modulus *G* of 70 GPa. The deflection takes into account both the bending and the shearing of the beam and there is also no transverse bending.

| Moment of inertia for an I-beam with different thickness of alumina | | | |
| --- | --- | --- | --- |
| I for 200nm | I for 400 nm | I for 600 nm | I for 800 nm |
| 7.809E-24 | 1.6104E-23 | 2.497E-23 | 3.4481E-23 |

Table S4: Deflection of the center point (in meters) under three-point bending with a pressure of 100 kPa applied to the spacer rib wall.

|  |  | Alumina thickness (nm) | | | |
| --- | --- | --- | --- | --- | --- |
|  |  | 200 | 400 | 600 | 800 |
| Length between contact points (µm) | 1 | 1.70E-09 | 8.52E-10 | 5.68E-10 | 4.26E-10 |
|  | 3 | 1.60E-08 | 7.98E-09 | 5.31E-09 | 3.98E-09 |
|  | 5 | 4.79E-08 | 2.39E-08 | 1.58E-08 | 1.18E-08 |
|  | 7 | 1.04E-07 | 5.18E-08 | 3.43E-08 | 2.55E-08 |
|  | 9 | 1.95E-07 | 9.66E-08 | 6.38E-08 | 4.74E-08 |
|  | 11 | 3.34E-07 | 1.65E-07 | 1.09E-07 | 8.04E-08 |
|  | 13 | 5.37E-07 | 2.65E-07 | 1.74E-07 | 1.28E-07 |
|  | 15 | 8.26E-07 | 4.06E-07 | 2.66E-07 | 1.96E-07 |
|  | 17 | 1.22E-06 | 6.00E-07 | 3.93E-07 | 2.88E-07 |
|  | 19 | 1.76E-06 | 8.60E-07 | 5.62E-07 | 4.12E-07 |
|  | 21 | 2.45E-06 | 1.20E-06 | 7.83E-07 | 5.73E-07 |
|  | 23 | 3.35E-06 | 1.64E-06 | 1.07E-06 | 7.80E-07 |
|  | 25 | 4.49E-06 | 2.19E-06 | 1.42E-06 | 1.04E-06 |


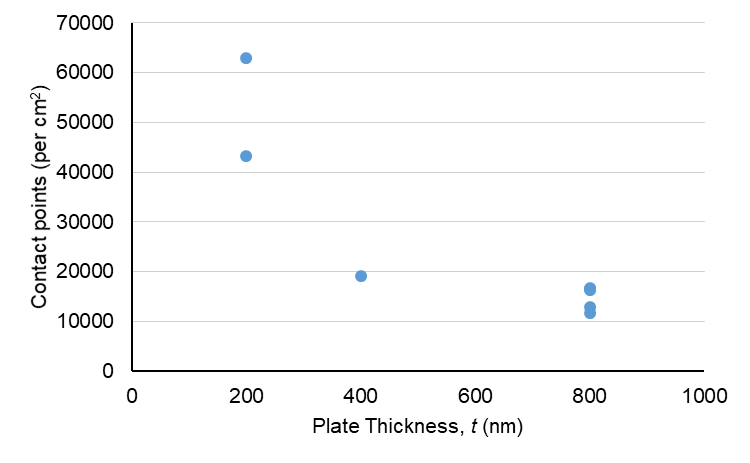


Figure S6: Plot of the number of sparse contact points versus the plate thickness based on the presented model for the data in Figure 5a of the main text. Due to the reduced stiffness and increased thermal resistance, it is likely that spacers with lower thickness had more points in contact for a given area.

Supplemental References

1. Davami, K. *et al.* Ultralight shape-recovering plate mechanical metamaterials. *Nature Communications* **6,** 10019 (2015).

2. Ylivaara, O. M. E. *et al.* Aluminum oxide from trimethylaluminum and water by atomic layer deposition: The temperature dependence of residual stress, elastic modulus, hardness and adhesion. *Thin Solid Films* **552,** 124–135 (2014).

3. Madhusudana, C. V. *Thermal Contact Conductance*. (Springer-Verlag, 1996).

4. Tsukizoe, T. & Hisakado, T. On the Mechanism of Contact Between Metal Surfaces—The Penetrating Depth and the Average Clearance. *J. Basic Eng* **87,** 666–672 (1965).

5. Tsukizoe, T. & Hisakado, T. On the Mechanism of Contact Between Metal Surfaces: Part 2—The Real Area and the Number of the Contact Points. *J. of Lubrication Tech* **90,** 81–88 (1968).

6. Greenwood, J. A. The Area of Contact Between Rough Surfaces and Flats. *Journal of Lubrication Technology* **89,** 81 (1967).

7. Greenwood, J. A. & Tripp, J. H. The Contact of Two Nominally Flat Rough Surfaces. *Proceedings of the Institution of Mechanical Engineers* **185,** 625–633 (1970).

8. Kimura, Y. Estimation of the number and the mean area of real contact points on the basis of surface profiles. *Wear* **15,** 47–55 (1970).

9. Mikić, B. B. Thermal contact conductance; theoretical considerations. *International Journal of Heat and Mass Transfer* **17,** 205–214 (1974).

10. ASME B46.1 - Surface Texture (Surface Roughness, Waviness, and Lay).

11. Cappella, A. *et al.* High Temperature Thermal Conductivity of Amorphous Al2O3 Thin Films Grown by Low Temperature ALD. *Advanced Engineering Materials* **15,** 1046–1050 (2013).
